# Blood pressure and age-related GFR decline in the general population

**DOI:** 10.1186/s12882-017-0496-7

**Published:** 2017-02-28

**Authors:** Bjørn O. Eriksen, Vidar T. N. Stefansson, Trond G. Jenssen, Ulla D. Mathisen, Jørgen Schei, Marit D. Solbu, Tom Wilsgaard, Toralf Melsom

**Affiliations:** 1 Metabolic and Renal Research Group, UiT The Arctic University of Norway, Tromsø, Norway; 2 Department of Community Medicine, Faculty of Health Sciences, UiT The Arctic University of Norway, Tromsø, Norway; 3 Section of Nephrology, University Hospital of North Norway, Tromsø, 9038 Norway; 4 Section of Nephrology, Department of Transplant Medicine, Oslo University Hospital, Rikshospitalet, Norway

**Keywords:** Age, Cardiovascular, Chronic renal failure, Elderly, Epidemiology, Hypertension

## Abstract

**Background:**

Hypertension is one of the most important causes of end-stage renal disease, but it is unclear whether elevated blood pressure (BP) also accelerates the gradual decline in the glomerular filtration rate (GFR) seen in the general population with increasing age. The reason may be that most studies have considered only baseline BP and not the effects of changes in BP, antihypertensive treatment and other determinants of GFR during follow-up. Additionally, the use of GFR estimated from creatinine or cystatin C instead of measurements of GFR may have biased the results because of influence from non-GFR related confounders. We studied the relationship between BP and GFR decline using time-varying variables in a cohort representative of the general population using measurements of GFR as iohexol clearance.

**Methods:**

We included 1594 subjects aged 50 to 62 years without baseline diabetes, kidney-, or cardiovascular disease in the Renal Iohexol-clearance Survey in Tromsø 6 (RENIS-T6). GFR, BP, antihypertensive medication and all adjustment variables were ascertained at baseline, and at follow-up after a median observation time of 5.6 years in 1299 persons (81%). The relationship between GFR decline and BP was analyzed in linear mixed models.

**Results:**

The mean (standard deviation) GFR decline rate was 0.95 (2.23) mL/min/year. The percentage of persons with hypertension (systolic BP ≥ 140 mmHg, diastolic BP ≥ 90 mmHg or antihypertensive medication) increased from 42 to 52% between baseline and follow-up. In multivariable adjusted linear mixed models using time-varying independent variables measured at baseline and follow-up, higher systolic and diastolic BP were associated with slower GFR decline rates by 0.10 and 0.20 mL/min/year/10 mmHg, respectively (*p* < 0.05). The association was stronger in persons on antihypertensive medication than in others (*p* < 0.05 for the interaction between BP and antihypertensive medication).

**Conclusions:**

In the medium-term, elevated BP is not associated with accelerated GFR decline in the general middle-aged population. In persons using antihypertensive medication, elevated BP is associated with a paradoxical slower GFR decline. Studies with even longer observation periods are needed to evaluate the ultimate effect of BP on kidney function.

**Electronic supplementary material:**

The online version of this article (doi:10.1186/s12882-017-0496-7) contains supplementary material, which is available to authorized users.

## Background

Hypertension is one of the most important causes of end-stage renal disease [1–6]. However, it is not clear whether hypertension also contributes to the gradual loss of kidney function seen in the general population with increasing age. The age-related decline in glomerular filtration rate (GFR) is the most important predisposing cause of chronic kidney disease (CKD) in old age, which affects 70% at eighty years of age [7]. Several observational studies have found an association between baseline blood pressure (BP) and subsequent GFR decline or incident CKD, [8–14]. but there are also studies that have shown no relationship or even a higher GFR [15–20]. In a recent meta-analysis of randomized controlled trials including 44,389 participants, intensified antihypertensive treatment had no statistically significant effect on the risk of end-stage renal disease [21]. Except in patients with diabetes or CKD, there is currently no evidence from randomized controlled trials that antihypertensive treatment or intensified antihypertensive treatment lowers the risk of kidney dysfunction [22–25].

There are several possible explanations for the divergent results of observational studies. One important explanation could be that the great majority of these investigations have studied GFR decline as an effect of BP and antihypertensive treatment at baseline. Since both BP and treatment are dynamic parameters, they will change during follow-up and exert a variable influence on GFR. Because the effect of BP on GFR is commonly believed to be continuous and not limited to a one-time baseline event, this approach may give a distorted picture of the relationship between them. To our knowledge, no previous study of age-related GFR decline in the general population has considered this issue. Additionally, previous studies have relied on GFR estimated from creatinine or cystatin C instead of actual measurements of GFR. These estimates are known to be influenced by several non-GFR factors that may have biased the results [26–28].

We have measured GFR as iohexol clearance in the Renal Iohexol-clearance Survey in Tromsø 6 (RENIS-T6) and again in the RENIS Follow-up Study (RENIS-FU) after a median observation time of 5.6 years. Iohexol clearance is recognized as a precise method for measuring GFR [29]. As far as we know, this is the only study of age-related GFR decline using GFR measurements. In a previous analysis, we found no association between baseline BP and GFR decline [30]. In the present study, we investigated the relationship between the GFR decline rate and BP and antihypertensive treatment ascertained as time-varying variables at both baseline and follow-up.

## Methods

### Study population

**Fig. 1:**
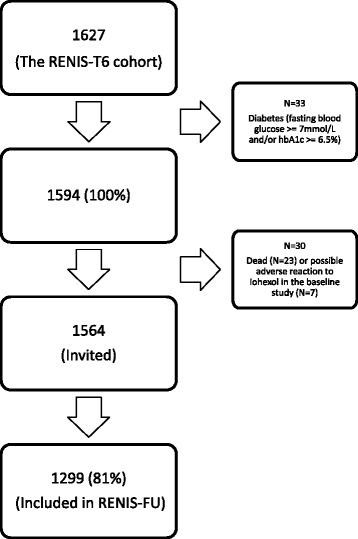
Inclusion of subjects in the RENIS follow-up study (RENIS-FU)

This study is a follow-up of RENIS-T6 which measured baseline iohexol-clearance in 1627 people between 50 and 62 years of age between 2007 and 2009. We included a representative sample of subjects from the general population without self-reported kidney disease, myocardial infarction, stroke or diabetes from the municipality of Tromsø in Northern Norway, as previously described in detail [31]. In the present study, we excluded 33 subjects who satisfied biochemical criteria for diabetes (fasting glucose ≥ 7.0 mmol/L and/or hemoglobin A1c ≥ 6.5%) at baseline, leaving 1594 subjects. Ten subjects with missing data for baseline hemoglobin A1c who all had fasting glucose < 7.0 mmol/L, were not excluded. Follow-up measurements of GFR in RENIS-FU were performed between September 2013 and January 2015. All the participants of the baseline study were invited except for 23 subjects who had died and 7 who had suffered a possible delayed allergic reaction to iohexol, leading to 1564 total people eligible (Fig. 1). A random sample of 5% was invited to a repeated follow-up GFR measurement to obtain a group of subjects with three GFR measurements, which is necessary for analysis with a linear mixed regression model with random intercept and slope and an unstructured covariance matrix [32].

This study complied with the Declaration of Helsinki and was approved by the Norwegian Data Inspectorate and the Regional Committee for Medical and Health Research Ethics of North Norway. All subjects provided written consent. All procedures were in accordance with institutional guidelines.

### Data

Both RENIS-T6 and RENIS-FU were conducted in the Clinical Research Unit at the University Hospital of North Norway. A health questionnaire with questions about alcohol and tobacco use and all current medications was administered on both occasions. Tobacco use was coded as the number of cigarettes currently smoked daily. Alcohol use was coded as 1 for the use of alcohol more than once a week and 0 otherwise.

### Measurements

#### Iohexol clearance

Baseline and follow-up GFR were measured using single-sample plasma clearance of iohexol, which has been validated against gold standard methods [33–36]. RENIS-FU used the same procedure as in the baseline RENIS-T6, which has previously been described in detail [30, 31].

A 6% random sample of blood samples from the baseline investigation previously frozen at −80 °C were thawed and reanalyzed to adjust for a possible drift in the method between baseline and follow-up. The mean difference in the GFR between follow-up and baseline was 2.28 mL/min/1.73 m^2^. All baseline GFR measurements reported in this study were adjusted by adding this constant to the original measurements, as described previously [30].

A total of 87 subjects had a repeated follow-up measurement of GFR. The mean coefficient of variation (95% confidence interval) for the intra-individual GFR variation was 4.2% (3.4–4.9%) [30]. The repeated follow-up measurements was performed a median (interquartile range (IQR)) of 35 (22–49) days after the first.

#### Blood pressure measurements

Attended BP measurements were performed by trained study nurses in the seated position after 2 min rest using an automated device and the appropriate cuff size (model UA 799; A&D, Tokyo, Japan). BP was measured three times with 1 min between measurements. The average of the second and the third measurement was used in the analyses. Hypertension was defined as systolic BP (SBP) ≥ 140 mmHg, diastolic BP (DBP) ≥ 90 mmHg or the use of antihypertensive medication. Mean pulse pressure (PP) was defined as SBP minus DBP and mean arterial pressure (MAP) as DBP plus one-third of the PP.

#### Other measurements

Fasting serum glucose, creatinine, cystatin C, triglycerides, and LDL- and HDL-cholesterol, hemoglobin A1C and the urine albumin-creatinine ratio (ACR) were measured with standard methods as described previously [37, 38]. Serum creatinine was measured using an enzymatic assay standardized to the isotope dilution mass spectroscopy method (CREA Plus, Roche Diagnostics, GmbH, Mannheim, Germany). Cystatin C was measured by a particle-enhanced turbidimetric immunoassay (Gentian, Moss, Norway) and calibrated to the international reference ERM-DA471/IFCC as previously described [39]. Estimated GFR (eGFR) was calculated from creatinine or cystatin C using the Chronic Kidney Disease Epidemiology Collaboration equations (eGFR_crea_ and eGFR_cys_) [40].

### Statistical methods

Mean (standard deviation (SD)) or median (IQR) for skewed variables were used for descriptive statistics. Differences between baseline and follow-up were tested with the paired *t*-test or Wilcoxon signed-rank test for continuous variables and McNemar’s test for paired dichotomous variables.

The GFR measurements were analyzed in linear mixed models with random intercept and slope [32, 41]. All of the subjects included in the baseline RENIS cohort were included in the linear mixed regression analyses regardless of whether they were examined at follow-up because linear mixed models allow for missing observations at one or more points in time [41, 42]. The subjects had from one to three GFR measurements: baseline (*n* = 1594), follow-up (*n* = 1299) and/or repeated follow-up (*n* = 87). Absolute GFR in mL/min was used as the dependent variable. Observation time from baseline was used as the independent time variable. Associations between the BP components (SBP, DBP, PP and MAP) and the rate of change in GFR were estimated by including two-way interaction terms between the BP variable in question and the time variable. Separate regression analyses were performed for each BP component. The linear mixed regression used time-varying values measured at both baseline and follow-up for all the independent variables, including BP components, antihypertensive treatment and adjustment variables.

The linear mixed regression analyses were adjusted for baseline age, sex and the following two sets of time-varying adjustment variables: Model 1 (body weight; height; individual dichotomous variables for the use of ACE-inhibitors, A2-receptor blockers, beta-blockers, calcium-blockers, diuretics and other antihypertensives) and model 2 (same variables as model 1 and also including LDL-cholesterol, HDL-cholesterol, fasting triglycerides, fasting glucose, ACR, pulse frequency, number of cigarettes currently smoked, and a dichotomous variable for the alcohol use).

Non-linear effects of the BP components on the GFR rate of change were explored by including second-degree fractional polynomial transformations of the BP components in the interactions with time in the linear mixed regression models [43].

Subjects with missing data for alcohol use (*n* = 6), ACR (*n* = 5) or triglycerides (*n* = 4) at baseline, and one subject with missing data for alcohol use at follow-up were excluded from the analyses. There were no missing data for the other independent variables or for GFR.

The same linear mixed regression analyses as described above were performed with change in estimated GFR assessed by eGFR_crea_ or eGFR_cys_ as the dependent variables. Statistical significance was set at 0.05. All of the statistical analyses were performed in STATA/MP 13.1 (www.stata.com).

## Results

A total of 1299 (81%) of the 1594 participants in the baseline cohort were investigated at follow-up after a median (IQR) observation time of 5.63 years (5.23–6.03) (Fig. 1). Most variables changed between baseline and follow-up (Table 1). The percentage of subjects with hypertension increased from 42 to 52 and the percentage of subjects receiving antihypertensives from 18 to 31 between baseline and follow-up. There was a slight increase in SBP and a slight reduction in DBP (*p* < 0.05). Except for the percentage of current smokers (18 vs. 28, *p* < 0.05), there were only small differences in baseline characteristics between those included and those lost to follow-up, as reported previously [30].

**Table 1:** Study population characteristics at baseline and follow-up. The RENIS-FU study

	Baseline	Follow-up	*P*-value for difference^a^
N (%)	1594	1299	
Male gender, n (%)	781 (49%)	643 (49%)	
Age, years	58.1 (3.8)	63.6 (4.0)	
Body mass index, kg/m2	27.2 (4.0)	27.1 (4.0)	0.59
Hypertension^b^, n (%)	674 (42%)	672 (52%)	<0.001
Systolic BP, mmHg	129.4 (17.5)	130.5 (16.9)	0.001
Diastolic BP, mmHg	83.4 (9.8)	81.9 (9.3)	<0.001
Pulse pressure, mmHg	46.1 (11.4)	48.6 (12.2)	<0.001
Mean arterial BP, mmHg	98.7 (11.7)	98.1 (10.9)	0.08
Pulse frequency, beats/min	66.6 (9.8)	64.5 (9.2)	<0.001
Antihypertensive medication, n (%)	289 (18%)	405 (31%)	<0.001
ACE inhibitor, n (%)	28 (1.8%)	48 (3.7%)	<0.001
A2 blocker, n (%)	132 (8.3%)	201 (15.5%)	<0.001
Betablocker, n (%)	67 (4.2%)	93 (7.2%)	<0.001
Calcium blocker, n (%)	80 (5.0%)	126 (9.7%)	<0.001
Diuretic, n (%)	140 (8.8%)	203 (15.6%)	<0.001
Other antihypertenives, n (%)	1 (0.1%)	5 (0.4%)	0.06
Current smoker, n (%)	322 (20%)	173 (13%)	<0.001
Use of alcohol more than 2–4 times a month, n (%)	434 (27%)	431 (33%)	0.01
LDL cholesterol, mmol/L	3.67 (0.86)	3.58 (0.90)	<0.001
HDL cholesterol, mmol/L	1.54 (0.42)	1.63 (0.46)	<0.001
Fasting triglycerides, mmol/L	1.00 (0.80 to 1.50)	1.00 (0.80 to 1.30)	0.12
Fasting glucose, mmol/L	5.30 (5.00 to 5.60)	5.40 (5.10 to 5.80)	<0.001
Urinary albumin-creatinine ratio, mg/mmol	0.23 (0.10 to 0.54)	0.34 (0.10 to 0.58)	<0.001
Absolute GFR, mL/min	103.8 (19.9)	98.2 (19.8)	<0.001
GFR, mL/min/1.73 m2	93.8 (14.3)	88.9 (14.5)	<0.001

Estimates are given as mean (standard deviation), median (interquartile range) or percent

*Abbreviations*: *RENIS-FU Study* the Renal Iohexol-clearance Survey Follow-up Study, *HDL* high-density lipoprotein, *LDL* low-density lipoprotein, *BP* blood pressure, *GFR* glomerular filtration rate

^a^Paired statistical tests for those who participated both at baseline and follow-up

^b^Systolic BP > = 140, diastolic BP > = 90 or antihypertensive medication

The unadjusted mean (SD) rate of change for the absolute GFR in the study period was −0.95 (2.23) mL/min/year. A negative change signifies a decline in GFR.

The absence of associations between baseline BP components and the GFR decline rate has been reported previously [30]. When analyzing time-varying BP with adjustment for independent variables measured at both baseline and follow-up; SBP, DBP and MAP, but not PP, were positively associated with GFR change in separate models, indicating slower GFR decline for higher BP values (*p* < 0.05) (Table 2), i.e. that lowering of BP was associated with a steeper GFR decline. Because time-varying independent variables for antihypertensive medication were used, these associations were independent of both the antihypertensive medication and changes in medication use between baseline and follow-up.

**Table 2:** The associations between time-varying blood pressure and GFR change rates in linear mixed regression analyses. The RENIS-FU study

BP component	Model 1^a^			Model 2^b^		
	Beta (mL/min/year)	95% confidence interval	*P*-value	Beta (mL/min/year)	95% confidence interval	*P*-value
Systolic BP per 10 mmHg	0.09	0.02 to 0.17	0.02	0.10^‡^	0.02 to 0.18	0.02
Diastolic BP per 10 mmHg	0.16	0.02 to 0.30	0.03	0.20^§^	0.05 to 0.34	0.01
Pulse pressure per 10 mmHg	0.08	-0.03 to 0.19	0.15	0.07	-0.04 to 0.18	0.21
Mean arterial pressure per 10 mmHg	0.15	0.03 to 0.27	0.01	0.17^||^	0.05 to 0.30	0.007

Each horizontal section in the table corresponds to one linear mixed regression model. Negative coefficients indicate a steeper GFR decline; positive coefficients a slower decline. The models used time-varying independent variables measured at both baseline and follow-up

*Abbreviations*: *RENIS-FU Study* the Renal Iohexol-clearance Survey Follow-up Study, *BP* blood pressure

^a^Model 1 adjusted for age; sex; body weight; height; individual dichotomous variables for the use of ACE-inhibitors, A2-receptor blockers, beta-blockers, calcium-blockers, diuretics and other antihypertensives

^b^Adjusted as model 1 and in addition LDL-cholesterol, HDL-cholesterol, fasting triglycerides, fasting glucose, urinary ACR, pulse frequency, number of cigarettes currently smoked, a dichotomous variable for the weekly use of alcohol or not

^‡^ *P* < 0.001 for the interaction between systolic BP and the use of any antihypertensive medication. Beta = 0.01 without and 0.33 mL/min/year/10 mmHg with antihypertensive medication

^§^ *P* = 0.001 for the interaction between diastolic BP and the use of any antihypertensive medication. Beta = 0.09 without and 0.42 mL/min/year/10 mmHg with antihypertensive medication

^||^ *P* < 0.001 for the interaction between mean arterial pressure and the use of any antihypertensive medication. Beta = 0.05 without and 0.49 mL/min/year/10 mmHg with antihypertensive medication

There were no statistically significant non-linear relationships between the BP components and GFR rate of change.

**Fig. 2:**
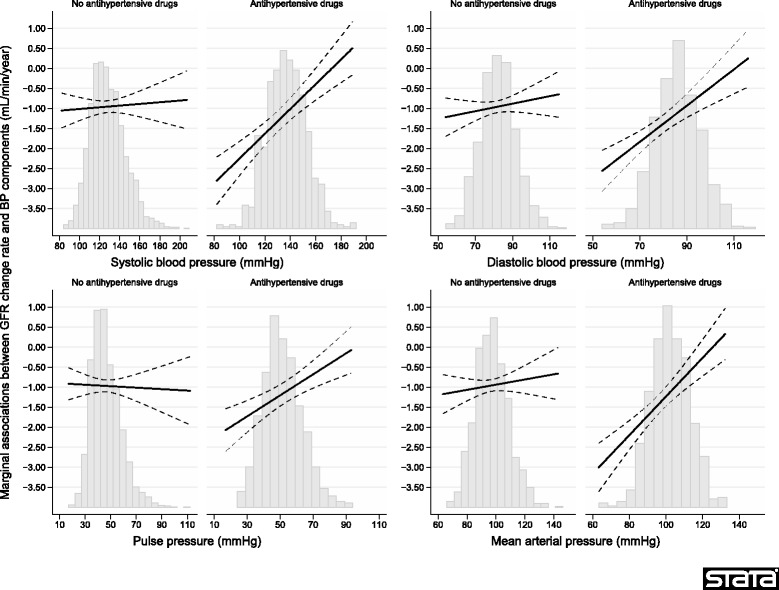
Associations between blood pressure components and GFR change rates in linear mixed models with time-varying independent variables. Separate curves for marginal GFR change rates with and without antihypertensive medication are shown (*p* < 0.05 for the interaction with antihypertensive medication for each blood pressure component). Dashed lines indicate 95% confidence intervals. Each curve should be interpreted as giving the marginal GFR change rate for a person with constant antihypertensive medication and BP component throughout the study period. The analyses were adjusted using time-varying variables for age, sex, body weight, height, LDL-cholesterol, HDL-cholesterol, fasting triglycerides, fasting glucose, urinary ACR, pulse frequency, number of cigarettes currently smoked, and a dichotomous variable for the weekly use of alcohol. The distribution of each blood pressure component is superimposed on each graph

There were statistically significant interactions between a dichotomous time-varying variable for antihypertensive treatment (yes/no) and SBP, DBP, and MAP respectively in the fully adjusted models in Table 2 (*p* < 0.001). The interactions indicate that the associations between GFR decline and SBP, DBP and MAP were stronger when combined with antihypertensive medication (Fig. 2).

We performed subgroup analyses for persons with hypertension at baseline and/or follow-up, for persons with normotension at both baseline and follow-up, for persons without self-reported heart disease, for persons without albuminuria (ACR less than 1.92 mg/mmol for men and 2.83 mg/mmol for women) and for persons with GFR greater than 60 ml/min/1.73 m^2^ (Additional file 1: Tables S1 and S2). The results were numerically similar to the main results in Table 2, but not statistically significant for SBP, DBP and MAP in the normotensive subgroup.

The associations between BP components and GFR decline assessed by body-surface adjusted GFR (GFR_BSA_) and estimated GFR based on either creatinine or cystatin C (eGFR_crea_ and eGFR_cys_) were analyzed (Additional file 1: Table S3). The same models as in Table 2 were used. There was no statistically significant relationship between any of the BP components and eGFR_crea_ or eGFR_cys_. The results for GFR_BSA_ were similar to those for absolute GFR in Table 2.

The regression models in Table 2 were re-analyzed with the mean of baseline and follow-up values substituted for the time-varying independent variables as predictors of the GFR decline slope. The results were essentially the same as in Table 2 (Additional file 1: Table S4).

## Discussion

The results of this investigation do not support the hypothesis that elevated BP causes accelerated age-related GFR decline in the general population during almost 6 years of follow-up (Table 2). On the contrary, the analyses indicate an association of higher SBP, DBP and MAP with a paradoxical slower GFR decline, i.e. that improved BP was associated with a steeper decline. There were statistically significant interactions between the BP components and antihypertensive treatment, so that the associations between BP and GFR decline occurred primarily in patients treated with antihypertensive agents (Fig. 2). The reason may be that GFR is more sensitive to BP changes in treated persons because long-standing hypertension or antihypertensive drugs interfere with the autoregulation of glomerular blood flow [44, 45]. This makes GFR more sensitive to changes in BP, which manifested as a steeper GFR decline with intensified BP treatment in our population.

In randomized controlled trials of antihypertensive drugs in CKD patients, an initial drop in GFR is commonly observed when treatment is started, followed by a subsequent slower decline [46, 47]. This finding has been interpreted as a beneficial effect of reducing an abnormally high GFR, so-called hyperfiltration [48]. Hyperfiltration is thought to be the first step in a sequence of events leading to reduced GFR and CKD [49]. However, there is also evidence of a longer-term drop in estimated GFR after intensive antihypertensive treatment, recently seen in two randomized trials with median follow-up of three years [24, 25]. Although our study population differs in several respects from the patients in these studies, our results suggest that the phase of reduced GFR due to an improvement of hyperfiltration may last longer than previously thought. This may explain the lack of effect on renal endpoint in trials with follow-up ranging from 1 to 7 years [22, 23].

To our knowledge, the present study is the first to assess the association between BP and GFR decline in the general population using measurements rather than estimates of GFR. Studies that have used serum creatinine, estimated GFR, or creatinine clearance have given mixed results [8–14, 50]. We found no statistically significant associations between BP and the change rates of estimated GFR based on creatinine and/or cystatin C (Additional file 1: Table S3). The reason may have been the influence of non-GFR factors on creatinine and cystatin C or the low precision of estimated GFR in the normal range [26–28]. To our knowledge, only one previous study of GFR decline has used time-varying BP in a non-CKD population [51]. Vupputuri et al. found an association between increased time-varying BP and rapid GFR decline. However, the study was not population-based, it used estimated GFR from creatinine and did not adjust for changes in antihypertensive medication. In the general population, we are not aware of any previous study that has adjusted for individual classes of antihypertensive medications or included time-varying adjustment variables. The considerable changes observed in both antihypertensive medication and hypertension status during the follow-up in our study underlines the importance of using statistical models that do not rely solely on baseline variables.

The finding of an association between higher BP and a slower GFR decline seems to be inconsistent with observational studies that have found hypertension a risk factor for both ESRD [1, 2, 5]. and less severe CKD [52–57]. One possible explanation is that additional genetic or environmental factors may lead to the development of progressive kidney disease in susceptible patients only. Another possibility is that renal hyperfiltration may be the first stage of hypertensive nephropathy, similar to the hyperfiltration phase of diabetic nephropathy. We measured whole kidney GFR which is a function of the total number of nephrons and single nephron GFR. Other studies have found a reduced nephron number in subjects with hypertension [58–60]. If we conservatively assume an average rate of age-related loss of nephrons in hypertensive subjects, a slower than average decline in whole kidney GFR implies an increasing single nephron GFR in the remaining nephrons. This means that our findings most likely reflect an elevated GFR or hyperfiltration at the single nephron level. Other studies have also found cross-sectional associations between elevated BP and hyperfiltration both in hypertensive patients [15–18]. and in the general population [19].

Conditions with both low BP and low GFR, such as advanced heart failure and serious infections, could possibly confound the association between low BP and a faster GFR decline, but are unlikely to explain the findings in this relatively healthy, ambulatory cohort. Only subjects without cardiovascular disease at baseline were included, and an analysis after excluding subjects with self-reported heart disease at follow-up made essentially the same findings as in the main analysis (Additional file 1: Table S1). We rescheduled GFR measurements for subjects with acute infections.

The principal strength of the present study is the use of measured GFR rather than estimated GFR from creatinine or cystatin C. When we repeated the analyses with estimated GFR instead of iohexol clearance, the regression coefficients were attenuated and not statistically significant (Additional file 1: Table S3). We limited confounding from comorbid conditions by excluding subjects with diabetes or cardiovascular disease at baseline. Because a third GFR measurement was obtained from a random subset of subjects, state-of-the-art linear mixed models rather than ordinary linear regression could be used for the analyses [61].

The most important limitation of our investigation was that conclusions about causality cannot be drawn from an observational study. In particular, the use of time-varying variables in the regression analyses does not allow us to establish the temporal precedence of elevated BP relative to a change in GFR decline, and can only demonstrate an association between these variables. The direction of causality is also uncertain, as subclinical renal damage has been suggested as a possible cause of primary hypertension [62]. In addition, the time varying variables were only ascertained at baseline and follow-up. More frequent measurements during follow-up would have given a more detailed picture of the relationship between BP and GFR and more precise estimates of effects, but would probably not have changed the overall conclusions or our study. Only middle-aged Caucasians were included and caution should be exercised when generalizing the results to other age groups and ethnic groups.

## Conclusions

We conclude that higher BP is not associated with an accelerated mean age-related GFR decline in the general population, but that lower BP because of antihypertensive treatment is associated with a medium-term steeper GFR decline. This finding is still consistent with a long-term beneficial effect of antihypertensive treatment on GFR decline, but indicates that a possible long term slowing effect on the decline may be preceded by a phase of reduced hyperfiltration of longer duration than previously thought. This should be investigated in studies with longer follow-up and repeated measurements of GFR. In primary hypertension, randomized controlled trials of the effect of antihypertensive treatment on renal endpoints should employ substantially longer follow-up than in previous trials.

## Additional file

Additional file 1: Four additional tables (**Table S1**-**S4**.) (DOCX 43 kb)

## Abbreviations

ACR: Albumin creatinine ratio
BP: Blood pressure
CKD: Chronic kidney disease
DBP: Diastolic BP
eGFR: Estimated glomerular filtration rate
eGFR_crea_: eGFR based on serum creatinine
eGFR_cys_: eGFR based on serum cystatin C
GFR: Glomerular filtration rate
GFR_BSA_: Body-surface adjusted GFR
IQR: Interquartile range
MAP: Mean arterial pressure
PP: Pulse pressure
RENIS-FU: RENIS Follow-up Study
RENIS-T6: Renal Iohexol-clearance Survey in Tromsø 6
SBP: Systolic BP
SD: Standard deviation
