# Supplementary material for: Blood pressure and age-related GFR decline in the general population
Source: BMC Nephrol. 2017 Feb 28;18:77. doi: 10.1186/s12882-017-0496-7 (PMC5331738; doi:10.1186/s12882-017-0496-7)
Supplement: Additional file 1: — Four additional tables (Table S1-S4.) (DOCX 43 kb) [file 12882_2017_496_MOESM1_ESM.docx]

**ADDITIONAL MATERIAL**

**BLOOD PRESSURE AND AGE-RELATED GFR DECLINE IN THE GENERAL POPULATION**

Bjørn O. Eriksen MD PhD^1,3^, Vidar T. N. Stefansson MD^1^, Trond G. Jenssen MD PhD^1,4^, Ulla D. Mathisen MD PhD^1,3^, Jørgen Schei MD^1^, Marit D. Solbu MD PhD^1,3^, Tom Wilsgaard PhD^2^, Toralf Melsom MD PhD^1,3^

^1^Metabolic and Renal Research Group, ^2^Dept. of Community Medicine, Faculty of Health Sciences, UiT The Arctic University of Norway; ^3^Section of Nephrology, University Hospital of North Norway, Tromsø; ^4^Section of Nephrology, Department of Transplant Medicine, Oslo University Hospital, Rikshospitalet, Norway

Correspondending author: Bjørn Odvar Eriksen, Section of Nephrology, University Hospital of North Norway, 9038 Tromsø, Norway

Fax: +4777628060; Telephone: +4777626856; E-mail: bjorn.odvar.eriksen@unn.no
